# Identification of Genes with Allelic Imbalance on 6p Associated with Nasopharyngeal Carcinoma in Southern Chinese

**DOI:** 10.1371/journal.pone.0014562

**Published:** 2011-01-20

**Authors:** Yan Li, Li Fu, Alissa Michelle Go Wong, Yan-Hui Fan, Miao-Xin Li, Jin-Xin Bei, Wei-Hua Jia, Yi-Xin Zeng, Danny Chan, Kenneth M. C. Cheung, Pak Sham, Daniel Chua, Xin-Yuan Guan, You-Qiang Song

**Affiliations:** 1 Department of Biochemistry, Li Ka Shing Faculty of Medicine, The University of Hong Kong, Hong Kong, Special Administrative Region, People's Republic of China; 2 Department of Clinical Oncology, Li Ka Shing Faculty of Medicine, The University of Hong Kong, Hong Kong, Special Administrative Region, People's Republic of China; 3 State Key Lab of Oncology in Southern China & Department of Experimental Research, Sun Yat-sen University Cancer Centre, Guangzhou, People's Republic of China; 4 Department of Orthopaedics and Traumatology, Li Ka Shing Faculty of Medicine, The University of Hong Kong, Hong Kong, Special Administrative Region, People's Republic of China; 5 Department of Psychiatry, Li Ka Shing Faculty of Medicine, The University of Hong Kong, Hong Kong, Special Administrative Region, People's Republic of China; 6 Centre for Reproduction, Development and Growth, Li Ka Shing Faculty of Medicine, The University of Hong Kong, Hong Kong, Special Administrative Region, People's Republic of China; Ohio State University Medical Center, United States of America

## Abstract

Nasopharyngeal carcinoma (NPC) is a malignancy of epithelial origin. The etiology of NPC is complex and includes multiple genetic and environmental factors. We employed case-control analysis to study the association of chromosome 6p regions with NPC. In total, 360 subjects and 360 healthy controls were included, and 233 single nucleotide polymorphisms (SNPs) on 6p were examined. Significant single-marker associations were found for SNPs rs2267633 (p = 4.49×10^−5^), rs2076483 (most significant, p = 3.36×10^−5^), and rs29230 (p = 1.43×10^−4^). The highly associated genes were the gamma-amino butyric acid B receptor 1 (*GABBR1*), human leukocyte antigen (*HLA-A*), and *HLA* complex group 9 (*HCG9*). Haplotypic associations were found for haplotypes AAA (located within *GABBR1*, p-value  = 6.46×10^−5^) and TT (located within *HLA-A*, p = 0.0014). Further investigation of the homozygous genotype frequencies between cases and controls suggested that micro-deletion regions occur in *GABBR1* and neural precursor cell expressed developmentally down-regulated 9 (*NEDD9*). Quantitative real-time polymerase chain reaction (qPCR) using 11 pairs of NPC biopsy samples confirmed the significant decline in *GABBR1* and *NEDD9* mRNA expression in the cancer tissues compared to the adjacent non-tumor tissue (p<0.05). Our study demonstrates that multiple chromosome 6p susceptibility loci contribute to the risk of NPC, possibly though *GABBR1* and *NEDD9* loss of function.

## Introduction

Nasopharyngeal carcinoma (NPC) is an epithelial-originated malignancy. Its etiology is complex and comprises multiple genetic and environmental factors. Epstein-Barr virus (EBV) infection [1], unhealthy lifestyle such as consumption of pickled food [2], and exposure to noxious inhalants [3] can predispose to NPC development. The incidence of NPC shows distinct geographical and ethnical differences. The susceptibility of NPC is nearly 100-fold higher in Southeast Asia, particularly in the Chinese province of Guangdong, than in most countries of predominantly European descent. Thus, NPC is also referred to as the Cantonese cancer because its incidence ranges from 10–50 cases per 100,000 people in this region [4], [5]. This NPC epidemic also shows familial aggregation; it was reported that the risk of NPC susceptibility is 9.31 times higher in first-degree relatives of the patient than in first-degree relatives of the spouse [6].

Genetic efforts to identify NPC susceptibility genes have employed both linkage studies and the candidate-gene–based approach [7]. Several chromosome regions, such as 3p12.3-14.2, 3q26.2-26.32 [8], 3p21.3-1-21.2 [9], and 6p21.3 [10], have been linked to NPC susceptibility and numerous disease-susceptible genes or loci have been associated with NPC. The chr6 super loci containing the human leukocyte antigen (HLA) system has been linked to the pathogenesis of NPC [10], [11], [12], as have the cell cycle regulation genes cyclin D1 (*CCND1*) [13], *p53* [14], and carcinogen-metabolism gene glutathione S-transferase M1 (*GSTM1*) [15]. More recently, two different groups utilized a genome-wide association study (GWAS) to scan the whole human genome for disease susceptibility loci [10], [12], and showed an elevated genetic susceptibility in southern Chinese.

The linkage of NPC to 6p21.3 [11] provides a genetic basis for a more thorough linkage analysis for disease susceptible loci in different populations. We began the study of NPC-associated genetic markers by using case-control analysis. The top 15 NPC genes within the linkage region were chosen from PubMed references (Table S2), and then tag single nucleotide polymorphisms (tag SNPs) within the genes were selected from the HapMap CHB database. In total, 233 tag SNPs ranging from 6095364–30048467 on chromosome 6p were selected to test whether the SNPs were associated with NPC in southern Chinese.

## Materials and Methods

### Study subjects and NPC biopsy samples

In total, 360 subjects of southern Chinese descent from Guangdong were pathologically diagnosed with NPC. Approvals for this study were obtained from the Hospital Institutional Review Board of the University of Hong Kong and Sun Yat-sen University Cancer Centre. A written informed consent was obtained from all participants. In the disease group, 71.8% were males, and 28.9% were females. The mean age of patients was 46.4±11.2 years (ranging from 16–75 years old). The healthy controls comprising 360 subjects were provided through the degenerate disc disease (DDD) study from southern Chinese (Area of Excellence Project from the University of Hong Kong). The controls were matched to the NPC cases by age (±5 years), sex, geographic region, and ethnicity. In the control group, 65.6% were females, and 34.4% were males. The mean age was 41.4±8.9 years (ranging from 14–63 years old).

### SNP selection

The SNPs in this study were selected based on the candidate gene in combination with PLINK and Haploview tag SNP selection algorithms. We used DDD study subjects as controls; the DDD study already has the whole genome scan data with 17,313 SNPs genotyped on chromosome 6. For quality control, the genotype missingness frequency (GENO) was set to GENO <0.05 and the minor allele frequency (MAF) to MAF >0.05 when we selected SNPs from the DDD study. After frequency and genotype pruning, 11,772 SNPs were left. Focusing solely on the genes located in the candidate region identified by the meta-analysis of the top candidate genes (PLINK filter from chromosome 6: 6–31 Mb), 2,730 SNPs remained, and among these only tag SNPs were selected using the HapMap tag SNP picking tool Tagger. The R-square cut-off value was set to 0.8. Only 233 tag SNPs were ultimately selected for genotyping.

### Sequenom® (San Diego, CA) SNP genotyping

The MassARRAY Assay Design software (Sequenom) was used to design amplification and allele-specific extension primers for uniplex or multiplexed assays. Polymerase chain reactions (PCR) were set up in 384-well plates with 6 uL total volume per reaction; the reaction mix contained 5 ng genomic DNA, 0.3 pmol each of the specific forward and reverse primers, 200 mM dNTP, 3.25 mM MgCl_2_, and 0.2 units of HotStar Taq polymerase (5 U/mL, Qiagen, Valencia, CA). The PCR conditions were as follows: initial denaturation at 95°C for 15 min, 45 cycles of 95°C for 20 s, 56°C for 30 s, and 72°C for 1 min, followed by final elongation at 72°C for 3 min. The extension primer was designed to hybridize to the amplicon located near the SNP site for the extension of a single base or a few bases depending on the genotype of the allele. Polymerase chain reactions, treatment of PCR products with alkaline phosphatase, and mass extension reactions were all performed according to the manufacturer's protocol (Sequenom). The final base-extension products were desalted using SpectroClean resin (Sequenom) mixed with 3-hydroxypicolinic acid, and analyzed using a modified Brucker Autoflex MALDI-TOF mass spectrometer (Brucker, Billerica, MA).

### ABI Taqman® SNP genotyping

The genotyping of 12 significant SNPs from a Taiwanese group was conducted using ABI Taqman (Carlsbad, CA) SNP genotyping assays. Human pre-designed Taqman probes were provided by the Taiwanese group (led by Prof. Chang). The assays were performed by ABI Prism 7900HT Sequence Detection System using the 384-well block at the Genome Research Centre, University of Hong Kong. Real-time data were analyzed using the SDS 2.3 application provided by ABI. Genotyping quality control was achieved through cross-plate controls and duplicates. Taqman SNP genotyping quality control was assessed by the service provider and checked manually. The average call rate per sample was >98%, whereas the average call rate per SNP was >95%.

### NPC tissues

To examine candidate gene expression, 20 primary NPC biopsies and adjacent normal tissue at the resection margins were collected immediately after the surgical resection at Queen Mary Hospital in Hong Kong. Of these samples, 11 provided enough high quality RNA for further investigation.

### NPC cell lines

The three NPC cell lines used in this study, CNE2 [16], SUNE1 [17], and C666-1 [18], were maintained in RPMI-1640 medium (Sigma, St. Louis, MO) and supplemented with 10% fetal bovine serum. An immortalized nasopharyngeal epithelial cell line, NP69 [19], was also cultured.

### Total RNA extraction and reverse transcription

Total RNA from the cell lines was extracted using Trizol reagent (Invitrogen, Carlsbad, CA) following the manufacturer's protocol. To maximize the yield of total RNA from small NPC biopsy samples, the total RNA was extracted using the miRVana PARIS kit (Ambion, Austin, TX) according to the manufacturer's protocol. Transcriptor High Fidelity cDNA Synthesis Kit (Roche Diagnostics Co., Indianapolis, IN, USA), prepared according to the manufacturer's protocol, was used to synthesize cDNA.

### Quantitative real-time PCR

For quantitative PCR (qPCR) analysis, cDNA were subjected to amplification with SYBR Green PCR Kit (Applied Biosystems, Foster City, CA, USA) using primers for neural precursor cell expressed, developmentally down-regulated 9 (*NEDD9*) and gamma-amino butyric acid B receptor 1 (*GABBR1*). Human 18s rRNA as used as the endogenous control. The threshold cycle (Ct) was determined in real time using an ABI PRISM 7700 Sequence Detector (Applied Biosystems, Foster City, CA, USA), and relative expression levels were analyzed using the 2-ΔΔCt method 2. Transcript quantification was performed in duplicate for each sample.

### Association and statistical analyses

The association analyses in this study were performed by PLINK (available online at: http://pngu.mgh.harvard.edu/purcell/plink/) [20]. Single SNP association analyses on allelic and genotypic associations include determining the p value, odds ratio (OR), and 95% confidence interval (CI). The Hardy-Weinberg equilibrium (HWE) and MAF were estimated by PLINK; for all tested SNPs, the HWE p-value was >0.001 and MAF was >0.01. Logistic regression analyses introduced age and gender as covariates. Linkage disequilibrium (LD) analysis was conducted using both PLINK [20] and Haploview 4.2 [21]. The panel of SNPs was entered into the Haploview software, and the individual haplotype frequency was estimated by the expectation-maximization (EM) algorithm. The haplotype structure was also analyzed by PLINK using the three-SNP sliding window option. Multiple testing was performed with 10,000 permutations and/or by Bonferroni correction. LocusZoom was used to generate the association plot (http://csg.sph.umich.edu/locuszoom/).

The detection of runs of homozygosity (ROH) analysis was done by PLINK. The algorithm involves taking a window of SNPs and sliding it across the region. The homozygosity is determined if the window looks “homozygous” enough at each window position. For each SNP, the proportion of “homozygous” windows that overlaps that position is calculated. The default window size and homozygous segment criteria were set to the following: 1,000 kb length, 100 SNPs, 50 kb/SNP density, and 1,000 kb for the largest gap [20].

The SPSS software standard version 13.0 (SPSS Inc., Chicago, IL) was used to perform the non-parametric t-tests, χ2 tests, and logistic regression tests to analyze the real-time PCR results quantitatively.

## Results

### Genetic association study of 6p SNPs in southern Chinese

**Figure 1:**
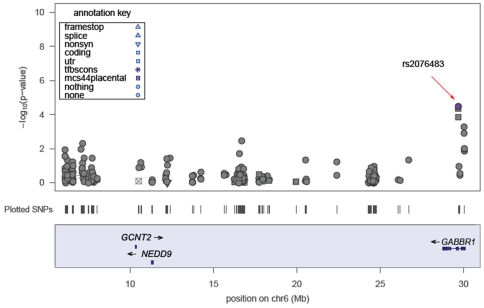
Single SNP allelic association analyses. The upper panel is the −log_10_ p-value plotted against the genomic location (LocusZoom), while the lower panel is the plotted single nucleotide polymorphisms (SNPs) of the studied region on chromosome 6p. Genes altered by micro-deletion(s) are depicted according to their physical locations and translation directions.

A genetic association study was performed on 360 NPC cases and 360 controls patients recruited of southern Chinese descent. For the 233 tag SNPs genotyped in this study, all followed the HWE in either the case or control group with MAF ≥0.05 (allelic association in Table S1). The overall genotyping call rate was ≥97.8%. Two markers (rs235158 and rs1997716) showed a very low informative rate (missing genotype >50%) and so were excluded from data analysis. Figure 1 shows the genotyped SNPs arranged according to their physical locations on Chr6 with allelic associations (−log_10_ p-values). The most significant association was found for SNP rs2076483 (p = 3.36×10^−5^). Two adjacent SNPs, rs2267633 (p = 4.49×10^−5^) and rs29230 (p = 1.43×10^−4^), located at the 6p23.31 region also showed high significance, suggesting that this region was significantly associated with NPC (Figure 1). The logistic regression test adjusted by age and sex did not change the associations. After correcting for multiple testing (Bonferroni correction), there were still significant associations for these three SNPs. The p-values were 0.0004 for rs2076483, 0.0005 for rs2267633, and 0.0017 for rs29230.

We noticed that the three most significant SNPs mapped to 6p23.31 region and were located within *GABBR1*, *HLA-A*, and *HCG9*. It was only last year that the Taiwan GWAS study reported these genes to be associated with NPC risk Thus, we were surprised that we could also detect the association with high significance by using only 360 patients/360 controls. This suggests that loci within the 6p23.31 region, and particularly *GABBR1* and *HLA-A* alleles confer high disease susceptibility. Further research on relevant functional genes and genetic replication in different populations should be conducted using a large dataset.

### Haplotype analysis of the *GABBR1* and *HLA-A* gene regions

This study involved over 200 SNPs located over a large region of 6p; thus, the pair-wise linkage disequilibrium (LD) analysis that was employed focused only on the regions and genes with high significance. Haplotype structure was analyzed using the Haploview software, and haplotype blocks were defined through confidence intervals (default). The most significantly associated haplotypes (AAA, p = 6.46×10^−5^ and GGG, p = 1.0×10^−4^, respectively) were located within *GABBR1* and comprised three significant SNPs, rs2267633, rs2076483, and rs29230. Haplotype AAA of *GABBR1* was present more frequently in the sample cases than in the controls (with frequency distributions of 0.822 and 0.733, respectively) and had a highly significant p-value of 6.46×10^−5^. This indicates that individuals carrying the AAA haplotype would be more susceptible to NPC than GGG carriers. In contrast, the haplotype GG composed of rs2517713 and rs2975042 within the *HLA-A* gene showed a protective effect against NPC (p = 7.0×10^−4^; case and control frequency distributions of 0.279 and 0.363), whereas the haplotype TT exhibited high risk for NPC disease (TT, p = 0.0014; case and control frequency distributions of 0.712 and 0.633). Multiple testing correction was conducted with 10,000 permutations; haplotypes AAA (p = 0.0008) and GGG (p = 0.0010) of *GABBR1* and haplotypes GG (p = 0.0072) and TT (p = 0.0134) of *HLA-A* all survived the multiple testing.

We then confirmed these findings by PLINK haplotype-association analysis. PLINK sliding window specification can determine haplotypes in the sliding windows of a fixed number of SNPs (shifting one SNP at a time) to form haplotypes across the entire dataset [20]. In 3-SNP sliding windows, haplotypes AAA and GGG formed by significant SNPs (rs2267633, rs2076483, and rs29230) reached statistical significance (p = 7.610×10^−5^ and p = 7.614×10^−5^ respectively). Two SNPs haplotypes formed by rs2517713 and rs2975042 were GG and TT, with p-values of 0.00078 and 0.00078 respectively, which were even more significant than Haploview results. Hence, haplotypic associations supported the single SNP association results and indicated the involvement of susceptibility genes in disease development.

### LOH and micro-deletions at 6p as detected by SNP genotyping

The high resolution of the SNP array and the large sample size enabled us to monitor the small DNA copy number changes occurring in NPC. In this study, the frequencies of homozygous genotypes were compared with those of heterozygous genotypes between NPC cases and matched healthy controls to detect frequent LOH loci at 6p, as performed in our previous study [22]. To identify the micro-deletions at 6p in NPC, the frequency of the homozygous genotype in the healthy controls and cases should be determined first. For each SNP marker, the ratio of homozygous frequency between the case and the control was calculated (T/N ratio). Using a threshold T/N ratio >1.0, 19 loci that reached statistical significance were considered frequent LOH loci. The micro-deleted region was defined when 3 or more adjacent SNP markers were considered frequent LOH loci. In this study, 3 micro-deletions were identified at 6p25-24, 6p21.31, and 6p21.3 (Table 1).

**Table 1:** Summary of frequent LOH loci at 6p detected by SNP-array.

SNP ID*	Location	T/N ratio^1^	*P* value	Gene name
rs2085575	6p25.3-24.3	1.2073538	0.004	F13A1
rs3024317	6p25.3-24.3	1.1449631	0.0181	F13A1
rs4960294	6p25	1.1457735	0.0298	RREB1
rs6597256	6p25	1.1318706	0.017	RREB1
rs267184	6p24-23	1.1431448	0.0253	BMP6
**rs504083**	6p24.2	1.1581754	0.0281	GCNT2
**rs1318748**	6p24.2	1.1529571	0.0371	GCNT2
**rs11759513**	6p25-24	1.1893557	0.0232	NEDD9
rs2137873	6p23	1.1861716	0.011	ATXN1
rs235147	6p23	1.1638418	0.03	ATXN1
rs236949	6p23	1.1905564	0.0047	ATXN1
rs2143083	6p22.3-22.2	1.3092179	0.0001	ALDH5A1
**rs2267633**	6p21.31	1.1809524	0.0033	GABBR1
**rs2076483**	6p21.31	1.2159952	0.0007	GABBR1
**rs29230**	6p21.31	1.1642882	0.0007	GABBR1
rs2517713	6p21.3	1.1925186	0.0109	HCP5P3
**rs9260734**	6p21.3	1.2099734	0.0033	HCG2P6
**rs3869062**	6p21.3	1.1733857	0.0101	HCG2P6
**rs5009448**	6p21.3	1.1841842	0.0164	MICD

1 T/N ratio  =  homozygous frequency ratio of cases/control.

* SNP markers at micro-deleted region in bold.

The presence of the extended homozygosity regions in human genome can influence the accuracy of SNP genotyping. These regions of homozygosity (ROHs) are characterized by multiple contiguous homozygous SNPs, with size ranging from 200 kb to more than 15 Mb [23], [24], [25]. To exclude interference by ROHs, we employed PLINK to screen for runs of homozygous genotypes. PLINK analyses showed that no ROH was detected across the studied region (chr6: 6,095,364–30,048,467). Genes located within the micro-deleted regions were then analyzed. The small deletions on 6p affected several genes, including Glucosaminyl (N-acetyl) transferase 2, I-branching enzyme (*GCNT2*), (*NEDD9*), and *GABBR1* (Table 1). The genes at 6p21.3 are pseudogenes, and thus they were not considered for further study. The *GCNT2* gene has never been linked to cancer development, so we selected *NEDD9* and *GABBR1* as the most promising potential candidate genes. We examined their mRNA expression levels in different cell lines and tissues.

### Examination of mRNA expression of candidate NPC susceptibility genes

**Figure 2:**
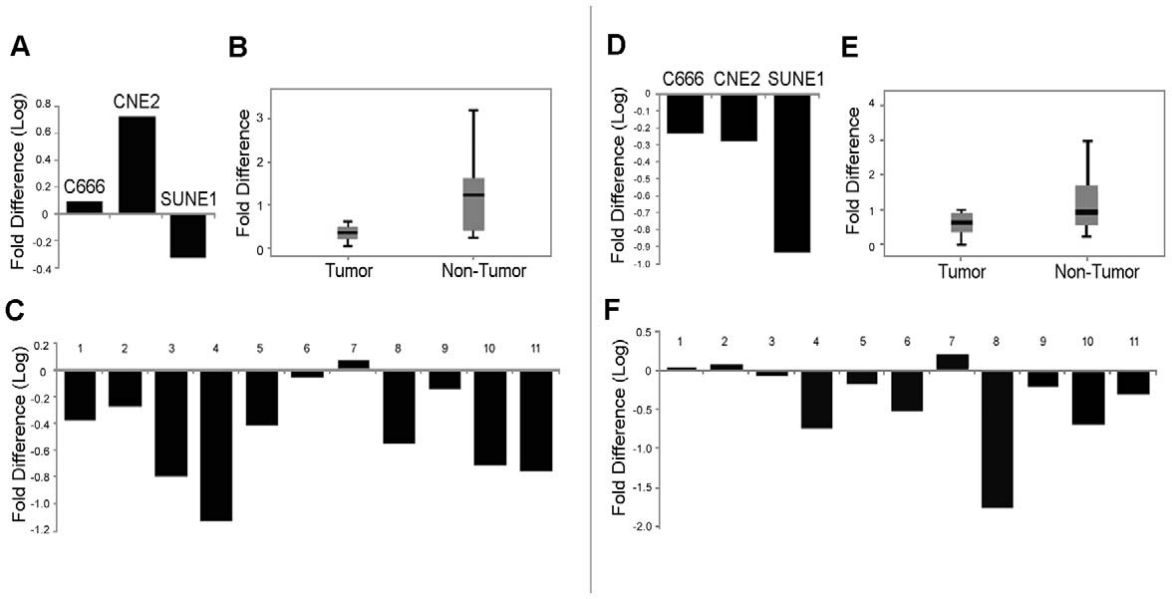
Expression of *NEDD9* (A–C) and *GABBR1* (D–F) at the transcription level. A and D are the real-time PCR results of *NEDD9* and/or *GABBR1* expressions in cell lines. Immortalized NP cell line NP69 was used as a control for gene expression in normal NP tissues. B and E are the boxplot diagrams of *NEDD9* and/or *GABBR1* expressions in 11 NPC tumor tissues and the adjacent non-tumor tissue. The difference in values was based on real-time PCR analysis normalized to endogenous 18s expression. C and F are the real-time PCR analyses of 11 pairs of NPC biopsy specimens. The changes in the *NEDD9* and/or *GABBR1* transcript expressions in each tumor specimen were compared against adjacent non-tumor tissues. P represents the paired t-test p-value.

To study the two candidate genes for the development of NPC, mRNA expression was characterized by quantitative real-time PCR in 3 NPC cell lines, CNE2, SUNE1, and C666-1, in the immortalized normal nasopharyngeal epithelial cell line NP69, and in 11 primary NPC tissue samples with adjacent normal tissue. Compared with the normal nasopharyngeal epithelial cell line NP69, SUNE1 and C666-1 cells demonstrated lower *NEDD9* mRNA expression. Conversely, the CNE2 cell line displayed an estimated 4-fold increase in *NEDD9* expression (Figure 2A). Compared with adjacent non-tumor tissue, 10 of 11 NPC tumor biopsy samples showed a significant down-regulation of *NEDD9* (P = 0.015; Figures 2B and 2C). Moreover, *GABBR1* was down-regulated in all three NPC cell lines (Figure 2D) and in 8/11 tumor biopsy tissues compared with the non-tumor tissue (Figures 2E and 2F). Statistically, the *GABBR1* gene showed a marginally significant association between the NPC tumor and non-tumor tissue specimens (P = 0.059). However, only 11 biopsy tissue samples were available. Confirmation of a link between the *GABBR1* gene and NPC will require larger sample sizes.

## Discussion

We demonstrated that multiple loci within 6p21.3 were associated with NPC susceptibility. This study used sample sets from southern China from which we found significant allelic and haplotype associations with NPC. Compared with the genome-wide association studies (GWASs) that examine large sample sets and thousands of genetic markers, the case-control analysis is often considered a necessary replication of GWAS findings. In this study, and in agreement with previous reports [10], [11], [12], the *HLA-A* region was significantly associated with NPC. We also found that the most significant SNPs were similar to those found in a Taiwanese GWAS [10]. However, unlike the results of this GWAS, the association in this study was at a SNP (rs2076483) adjacent to that isolated in the Taiwan group (rs29230), but within the same gene, of *GABBR1*. The subjects analyzed were all southern Chinese and the MAFs were similar; thus, such deviation might be due to genetic heterogeneity. None-the-less, that finding that both associated SNPs were within *GABBR1*underscores a possible role in the etiology of NPC.

Aside from genetic association, we further analyzed the SNP genotyping data to identify the micro-deleted regions at 6p (6p25-24, 6p21.31, and 6p21.3). Although SNP arrays are powerful tools for the identification of chromosomal aberrations, especially deletions or amplifications [26], there are several limitations to this approach. One technical limitation is that SNP assays are optimized for allelic discrimination rather than for copy number measurement. Another potential drawback is the presence of ROH, which could influence the accuracy of SNP genotyping. As all the tag SNPs were derived from the HapMap dataset, there is no clear indication of the existence of ROH across the studied region; likewise, the PLINK ROH analysis showed the absence of ROH within this region. Hence, it was possible to use SNP genotyping data to detect micro-deletions within the region of interest.

Two candidate genes located within the micro-deleted regions, *NEDD9* at 6p25-24 and *GABBR1* at 6p21.31, were absent or down-regulated at the mRNA expression level in primary NPC tumors and NPC cell lines. Although the sample size (comprising only 11 tumor samples) used for quantitative real-time PCR analysis was not sufficient for statistical calculation, the qPCR results did show alterations in gene expression levels. Here, the different expression changes were from the copy number variations in the tumor DNA and there was no direct link to the micro-deletion detected when we compared normal DNAs between cases and controls. However, the different expression levels of *NEDD9* and *GABBR1* between PNC tumors and normal tissues suggested the importance of both genes in PNC development. A population-based study to determine if micro-deletion in normal DNAs can also reduce *GABBR1* expression is clearly warranted.

The *NEDD9* gene, also known as *HEF1* and *CAS-L*, encodes a scaffolding protein that promotes tumor cell migration and invasion, but at the same time inducing apoptosis and mitotic defects that trigger cell cycle arrest [26], [27], [28]. The *NEDD9* protein product is subject to dynamic and complex regulation [29]. Indeed, *TGF*-β [30], retinoic acid [31], and progesterone receptor A [32] have all been reported to up-regulate the *NEDD9* transcription, whiles estrogen down-regulated *NEDD9* mRNA in osteosarcoma [33] and breast cancer cells [34]. This study provides the first evidence that the *NEDD9* gene is subject down-regulation at the transcriptional level due to copy number loss in NPC tumors.

The *GABBR1* gene was mapped to chromosome 6p21.31 within the *HLA* class I region that also contains susceptibility loci for multiple sclerosis, epilepsy, and schizophrenia. As a receptor component of the inhibitory GABAergic system that transduces GABA release into metabotropic signal transduction cascades, the up-regulation of *GABBR1* may have a suppressive effect on the behavior of malignant tumors in NPC cancer cells [35]. A recent study by Schuller et al. (2008) [36] demonstrated that *GABBR1* could inhibit beta-adrenoreceptors (β-AR) signaling in pancreatic cancer cells, thus blocking the driving forces of cancer progression such as cell proliferation and cell migration; this indicates the unexpected involvement of *GABBR1* in cancer genesis.

The Taiwan GWAS was the first study to associate *GABBR1* with NPC and found elevated *GABBR1* protein expression in NPC tumor tissues compared with the adjacent normal epithelial cells [10]. Interestingly, when the *GABBR1* transcript and protein levels in NPC cell lines were examined, down-regulation of *GABBR1* protein in two NPC cell lines, BM1 and HK1 (AA genotype at rs29232), was observed compared with the immortalized nasopharyngeal epithelial cell line NP69 (AG genotype at rs29232). The risk allele of rs29232 was “A,” and thus the homozygous carrier of A-allele showed a lower protein level than the heterozygous carrier. However, the study did not compare the *GABBR1* transcript and protein expression levels between normal and cancer cell lines. Thus, the relationship between A-allele-carrier and gene expression levels remains unknown. In this study, we reported the down-regulation of *GABBR1* transcripts in NPC tumors, indicating that down-regulation of gene expression might be one of the tumorigenic mechanisms. Given the importance of *GABBR1* involvement in NPC, its expression at the transcriptional and protein levels requires further research.

In the course of this study, another GWAS, which was conducted by a group from Guangzhou [12], was made available online. Bei et al. (2010) validated the strong associations within the HLA regions on 6p. In addition, they detected 3 new NPC susceptibility loci on 3q26, 9p21, and 13q12 and identified novel risk genes (*TNFRSF19*, *MDS1-EVI1*, and *CDKN2A-CDKN2B* gene clusters) [12]. Their analyses showed that other candidate genes and cancer genesis mechanisms could underlie the NPC pathogenic process. In view of the high prevalence of NPC in the southern China population, future study on NPC should focus on novel pathogenic loci to discover new tumorigenic gene and provide clinical targets for treatment.

## Supporting Information

Table S1 Single SNP association of NPC data set.
(0.44 MB DOC)

Table S2 Candidate genes located in “hot spots” identified by meta-analysis (6p21.2-p23).
(0.01 MB DOCX)
