# Supplementary material for: Identification of Genes with Allelic Imbalance on 6p Associated with Nasopharyngeal Carcinoma in Southern Chinese
Source: PLoS One. 2011 Jan 20;6(1):e14562. doi: 10.1371/journal.pone.0014562 (PMC3024318; doi:10.1371/journal.pone.0014562)
Supplement: Table S1 — Single SNP association of NPC data set. (0.44 MB DOC) [file pone.0014562.s001.doc]

Supplementary Table1 Single SNP association of NPC data set

| CHR | SNP | BP | A1 | F_A | F_U | A2 | CHISQ | P | OR |
| --- | --- | --- | --- | --- | --- | --- | --- | --- | --- |
| 6 | rs3778360 | 6095364 | T | 0.4132 | 0.3505 | C | 6.385 | 0.01151 | 1.305 |
| 6 | rs3778359 | 6095381 | A | 0.2813 | 0.232 | G | 4.879 | 0.02719 | 1.296 |
| 6 | rs6914953 | 6098442 | A | 0.09682 | 0.08656 | G | 0.4825 | 0.4873 | 1.131 |
| 6 | rs6928884 | 6107059 | G | 0.4337 | 0.3776 | A | 4.964 | 0.02587 | 1.262 |
| 6 | rs4416719 | 6109207 | T | 0.2147 | 0.1842 | A | 2.194 | 0.1386 | 1.211 |
| 6 | rs7769202 | 6114655 | G | 0.225 | 0.2192 | A | 0.07511 | 0.784 | 1.034 |
| 6 | rs11969912 | 6120974 | T | 0.3026 | 0.3085 | C | 0.06157 | 0.804 | 0.9727 |
| 6 | rs7774391 | 6121624 | C | 0.4109 | 0.4352 | T | 0.9237 | 0.3365 | 0.9051 |
| 6 | rs6597195 | 6121837 | A | 0.4868 | 0.4532 | T | 1.712 | 0.1907 | 1.145 |
| 6 | rs3778353 | 6124881 | G | 0.1631 | 0.1744 | A | 0.3445 | 0.5573 | 0.923 |
| 6 | rs3799559 | 6126034 | A | 0.5132 | 0.4808 | G | 1.615 | 0.2038 | 1.138 |
| 6 | rs3799557 | 6130016 | A | 0.2162 | 0.2163 | G | 4.47E-05 | 0.9947 | 0.9992 |
| 6 | rs2085575 | 6150841 | A | 0.2823 | 0.3165 | C | 2.137 | 0.1438 | 0.8494 |
| 6 | rs1742928 | 6171950 | G | 0.5013 | 0.4923 | T | 0.1249 | 0.7238 | 1.037 |
| 6 | rs1742929 | 6173024 | T | 0.1878 | 0.1724 | C | 0.6137 | 0.4334 | 1.11 |
| 6 | rs3851513 | 6219160 | G | 0.2143 | 0.1906 | A | 1.322 | 0.2502 | 1.158 |
| 6 | rs3851514 | 6219569 | T | 0.4496 | 0.4359 | C | 0.2903 | 0.59 | 1.057 |
| 6 | rs9328348 | 6220931 | C | 0.347 | 0.3865 | T | 2.59 | 0.1075 | 0.8434 |
| 6 | rs1267866 | 6228369 | T | 0.1777 | 0.1403 | C | 4.002 | 0.04545 | 1.325 |
| 6 | rs9502429 | 6229124 | A | 0.4489 | 0.4731 | G | 0.8939 | 0.3444 | 0.9074 |
| 6 | rs1267913 | 6236109 | C | 0.4722 | 0.4871 | T | 0.3383 | 0.5608 | 0.9422 |
| 6 | rs1937802 | 6248038 | A | 0.06711 | 0.06298 | G | 0.1075 | 0.743 | 1.07 |
| 6 | rs3024347 | 6248210 | T | 0.347 | 0.3232 | C | 0.9592 | 0.3274 | 1.113 |
| 6 | rs9405913 | 6248273 | C | 0.05867 | 0.05141 | T | 0.3867 | 0.5341 | 1.15 |
| 6 | rs3024345 | 6248297 | A | 0.2659 | 0.2584 | T | 0.1121 | 0.7378 | 1.04 |
| 6 | rs7758057 | 6252577 | C | 0.3303 | 0.3078 | G | 0.8891 | 0.3457 | 1.109 |
| 6 | rs1674043 | 6255615 | A | 0.1 | 0.1117 | T | 0.5519 | 0.4575 | 0.8837 |
| 6 | rs3024317 | 6264378 | C | 0.2676 | 0.2841 | T | 0.5163 | 0.4724 | 0.9207 |
| 6 | rs481924 | 6532057 | G | 0.4248 | 0.4182 | A | 0.06866 | 0.7933 | 1.028 |
| 6 | rs3804464 | 6536282 | G | 0.09788 | 0.1118 | A | 0.7933 | 0.3731 | 0.8618 |
| 6 | rs9328374 | 6536628 | C | 0.3289 | 0.3208 | T | 0.1149 | 0.7346 | 1.038 |
| 6 | rs3761985 | 6539030 | G | 0.1908 | 0.1624 | T | 2.138 | 0.1437 | 1.216 |
| 6 | rs9378475 | 6543883 | T | 0.1223 | 0.1061 | C | 0.9957 | 0.3183 | 1.174 |
| 6 | rs932364 | 6561589 | T | 0.1544 | 0.1517 | C | 0.02132 | 0.8839 | 1.021 |
| 6 | rs3804474 | 6566507 | G | 0.4895 | 0.4409 | A | 3.65 | 0.05606 | 1.216 |
| 6 | rs7770797 | 6566961 | C | 0.3577 | 0.3363 | T | 0.7747 | 0.3788 | 1.099 |
| 6 | rs1076673 | 6572183 | A | 0.1395 | 0.1617 | C | 1.454 | 0.2278 | 0.8401 |
| 6 | rs3827783 | 6574986 | C | 0.276 | 0.2391 | T | 2.725 | 0.09876 | 1.213 |
| 6 | rs2064236 | 6584424 | G | 0.3408 | 0.3522 | A | 0.2173 | 0.6411 | 0.9512 |
| 6 | rs9392818 | 6586034 | A | 0.1098 | 0.1133 | G | 0.0469 | 0.8285 | 0.9654 |
| 6 | rs3804489 | 6587794 | C | 0.4187 | 0.4284 | T | 0.1482 | 0.7003 | 0.961 |
| 6 | rs7772296 | 7059841 | T | 0.1398 | 0.09769 | C | 6.532 | 0.01059 | 1.502 |
| 6 | rs1285882 | 7101900 | G | 0.06614 | 0.05584 | A | 0.7062 | 0.4007 | 1.197 |
| 6 | rs9505057 | 7116406 | G | 0.3408 | 0.305 | T | 2.212 | 0.1369 | 1.178 |
| 6 | rs6935691 | 7123538 | A | 0.2797 | 0.2526 | T | 1.427 | 0.2322 | 1.149 |
| 6 | rs4959431 | 7142345 | A | 0.3734 | 0.4082 | G | 1.961 | 0.1614 | 0.8639 |
| 6 | rs4960294 | 7143972 | G | 0.2487 | 0.2896 | T | 3.248 | 0.07152 | 0.8119 |
| 6 | rs6597256 | 7153006 | G | 0.1601 | 0.2165 | A | 7.97 | 0.004755 | 0.6896 |
| 6 | rs2714348 | 7167812 | G | 0.2613 | 0.2378 | C | 1.128 | 0.2882 | 1.134 |
| 6 | rs2714338 | 7195959 | T | 0.4087 | 0.3939 | C | 0.3537 | 0.552 | 1.064 |
| 6 | rs9505117 | 7235281 | T | 0.4008 | 0.3731 | C | 1.244 | 0.2648 | 1.124 |
| 6 | rs7748736 | 7247034 | C | 0.464 | 0.4701 | T | 0.05584 | 0.8132 | 0.976 |
| 6 | rs2764091 | 7247415 | G | 0.1885 | 0.1478 | A | 4.522 | 0.03346 | 1.339 |
| 6 | rs2806167 | 7488782 | C | 0.3219 | 0.3432 | A | 0.7837 | 0.376 | 0.9085 |
| 6 | rs9392904 | 7500853 | G | 0.3886 | 0.389 | C | 0.0003092 | 0.986 | 0.9982 |
| 6 | rs3778337 | 7510884 | T | 0.4127 | 0.4269 | C | 0.3189 | 0.5723 | 0.9433 |
| 6 | rs2259208 | 7519804 | C | 0.4198 | 0.4286 | T | 0.121 | 0.728 | 0.9647 |
| 6 | rs2757592 | 7532193 | G | 0.2453 | 0.2206 | A | 1.295 | 0.2551 | 1.148 |
| 6 | rs7753111 | 7675943 | C | 0.2855 | 0.2668 | T | 0.6772 | 0.4106 | 1.099 |
| 6 | rs2876117 | 7683930 | A | 0.134 | 0.162 | C | 2.348 | 0.1254 | 0.801 |
| 6 | rs911749 | 7683958 | T | 0.2922 | 0.2982 | C | 0.06534 | 0.7982 | 0.9717 |
| 6 | rs270398 | 7710839 | G | 0.4602 | 0.4884 | T | 1.215 | 0.2704 | 0.8932 |
| 6 | rs1885448 | 7716777 | C | 0.3276 | 0.3329 | G | 0.04847 | 0.8257 | 0.9763 |
| 6 | rs11243204 | 7717698 | G | 0.1402 | 0.1801 | A | 4.503 | 0.03383 | 0.7426 |
| 6 | rs9392924 | 7740099 | G | 0.488 | 0.4576 | C | 1.422 | 0.233 | 1.13 |
| 6 | rs11964227 | 7765668 | T | 0.2316 | 0.2141 | C | 0.6791 | 0.4099 | 1.106 |
| 6 | rs267184 | 7778428 | C | 0.2613 | 0.2841 | T | 1.002 | 0.3168 | 0.8914 |
| 6 | rs267202 | 7799235 | C | 0.2267 | 0.2185 | T | 0.1469 | 0.7015 | 1.048 |
| 6 | rs9505293 | 7815592 | A | 0.1151 | 0.102 | C | 0.6768 | 0.4107 | 1.144 |
| 6 | rs1225933 | 7821214 | G | 0.3474 | 0.3458 | A | 0.004401 | 0.9471 | 1.007 |
| 6 | rs1225948 | 7835271 | G | 0.3386 | 0.3506 | A | 0.2442 | 0.6212 | 0.9481 |
| 6 | rs443861 | 7841490 | T | 0.1227 | 0.1328 | C | 0.3423 | 0.5585 | 0.9131 |
| 6 | rs9505356 | 8028977 | T | 0.4162 | 0.4321 | C | 0.3923 | 0.5311 | 0.937 |
| 6 | rs12526001 | 8036524 | C | 0.1127 | 0.1054 | G | 0.212 | 0.6452 | 1.078 |
| 6 | rs303050 | 10511169 | C | 0.2791 | 0.2739 | T | 0.05167 | 0.8202 | 1.026 |
| 6 | rs17635655 | 10526556 | T | 0.03979 | 0.05656 | A | 2.342 | 0.1259 | 0.6912 |
| 6 | rs504083 | 10649199 | T | 0.2842 | 0.3274 | C | 3.379 | 0.06601 | 0.8158 |
| 6 | rs1318748 | 10682298 | A | 0.3471 | 0.3862 | G | 2.525 | 0.1121 | 0.8449 |
| 6 | rs11759513 | 11306800 | A | 0.496 | 0.5013 | G | 0.04244 | 0.8368 | 0.9791 |
| 6 | rs9295813 | 11311011 | G | 0.1082 | 0.11 | A | 0.01276 | 0.9101 | 0.9817 |
| 6 | rs9357087 | 11324686 | T | 0.1359 | 0.1384 | C | 0.02008 | 0.8873 | 0.9791 |
| 6 | rs7769173 | 11332734 | A | 0.3995 | 0.388 | C | 0.2092 | 0.6474 | 1.049 |
| 6 | rs11970705 | 11337939 | T | 0.2202 | 0.2163 | C | 0.03293 | 0.856 | 1.023 |
| 6 | rs4714119 | 12127340 | T | 0.4524 | 0.4652 | A | 0.2537 | 0.6145 | 0.9497 |
| 6 | rs6925772 | 12128227 | A | 0.1892 | 0.2018 | G | 0.3898 | 0.5324 | 0.9227 |
| 6 | rs12525800 | 12143315 | A | 0.2413 | 0.2615 | C | 0.8262 | 0.3634 | 0.8984 |
| 6 | rs4714150 | 12162101 | A | 0.1979 | 0.1922 | C | 0.07854 | 0.7793 | 1.037 |
| 6 | rs9296253 | 12190675 | A | 0.09284 | 0.07623 | G | 1.363 | 0.243 | 1.24 |
| 6 | rs9369083 | 12207875 | C | 0.2566 | 0.261 | T | 0.03822 | 0.845 | 0.9774 |
| 6 | rs7768491 | 12210601 | C | 0.1923 | 0.1517 | T | 4.374 | 0.03648 | 1.331 |
| 6 | rs2228220 | 12231235 | C | 0.1154 | 0.1157 | T | 0.0003297 | 0.9855 | 0.9971 |
| 6 | rs7741589 | 12237033 | G | 0.4265 | 0.4698 | A | 2.867 | 0.09039 | 0.8391 |
| 6 | rs2070699 | 12400758 | C | 0.4684 | 0.5223 | A | 4.416 | 0.03559 | 0.8061 |
| 6 | rs3800503 | 13727608 | C | 0.3714 | 0.3756 | T | 0.03007 | 0.8623 | 0.9818 |
| 6 | rs9382303 | 13735130 | C | 0.4421 | 0.4648 | T | 0.7969 | 0.372 | 0.9123 |
| 6 | rs7760411 | 13736038 | T | 0.2779 | 0.2807 | C | 0.01429 | 0.9048 | 0.9864 |
| 6 | rs6458997 | 13758950 | T | 0.1609 | 0.1636 | C | 0.02152 | 0.8834 | 0.9798 |
| 6 | rs204226 | 13814229 | G | 0.4526 | 0.4769 | C | 0.9075 | 0.3408 | 0.9072 |
| 6 | rs3799924 | 14236320 | G | 0.137 | 0.1263 | A | 0.3842 | 0.5354 | 1.098 |
| 6 | rs9396472 | 14240866 | T | 0.4867 | 0.4562 | G | 1.408 | 0.2354 | 1.13 |
| 6 | rs853360 | 14242571 | A | 0.1698 | 0.1594 | G | 0.3001 | 0.5838 | 1.078 |
| 6 | rs13437303 | 15639583 | T | 0.07237 | 0.0874 | C | 1.181 | 0.2771 | 0.8146 |
| 6 | rs9396592 | 15646989 | G | 0.4259 | 0.4491 | A | 0.8293 | 0.3625 | 0.9102 |
| 6 | rs10949309 | 15758863 | T | 0.2691 | 0.291 | C | 0.9138 | 0.3391 | 0.8971 |
| 6 | rs2072783 | 16251876 | C | 0.3628 | 0.376 | T | 0.2862 | 0.5926 | 0.9451 |
| 6 | rs2072781 | 16255328 | C | 0.2473 | 0.2494 | T | 0.00833 | 0.9273 | 0.9892 |
| 6 | rs7763322 | 16362074 | A | 0.119 | 0.125 | G | 0.124 | 0.7247 | 0.9459 |
| 6 | rs179993 | 16429771 | T | 0.129 | 0.1478 | G | 1.135 | 0.2866 | 0.8538 |
| 6 | rs2237224 | 16429806 | T | 0.07733 | 0.08226 | C | 0.1263 | 0.7223 | 0.9351 |
| 6 | rs9396666 | 16482485 | G | 0.07353 | 0.06364 | A | 0.5819 | 0.4456 | 1.168 |
| 6 | rs2237219 | 16483231 | C | 0.4443 | 0.4301 | G | 0.3106 | 0.5773 | 1.059 |
| 6 | rs7738608 | 16492644 | C | 0.06782 | 0.06041 | T | 0.35 | 0.5541 | 1.132 |
| 6 | rs2237216 | 16496162 | T | 0.4946 | 0.4707 | C | 0.88 | 0.3482 | 1.101 |
| 6 | rs179944 | 16506117 | A | 0.4021 | 0.3706 | G | 1.563 | 0.2113 | 1.142 |
| 6 | rs3819405 | 16507536 | T | 0.321 | 0.3364 | C | 0.4096 | 0.5222 | 0.9324 |
| 6 | rs434905 | 16517476 | A | 0.05526 | 0.07308 | T | 2.031 | 0.1542 | 0.742 |
| 6 | rs9370893 | 16526541 | T | 0.3201 | 0.321 | C | 0.001324 | 0.971 | 0.996 |
| 6 | rs2237203 | 16532986 | C | 0.1878 | 0.2136 | A | 1.584 | 0.2081 | 0.8517 |
| 6 | rs932411 | 16533283 | T | 0.391 | 0.4023 | C | 0.206 | 0.6499 | 0.9537 |
| 6 | rs3806127 | 16549222 | A | 0.2685 | 0.262 | G | 0.083 | 0.7733 | 1.034 |
| 6 | rs2137873 | 16567620 | A | 0.296 | 0.3523 | G | 5.507 | 0.01895 | 0.7729 |
| 6 | rs17593746 | 16576283 | C | 0.186 | 0.1907 | T | 0.05335 | 0.8173 | 0.97 |
| 6 | rs1514328 | 16582288 | G | 0.1289 | 0.1382 | A | 0.2862 | 0.5927 | 0.9228 |
| 6 | rs7750263 | 16584907 | G | 0.4528 | 0.4293 | A | 0.8441 | 0.3582 | 1.1 |
| 6 | rs235146 | 16595223 | C | 0.2401 | 0.2474 | T | 0.1127 | 0.7371 | 0.9609 |
| 6 | rs235147 | 16595385 | A | 0.4051 | 0.4031 | G | 0.00648 | 0.9358 | 1.008 |
| 6 | rs2237189 | 16596335 | A | 0.2143 | 0.1987 | G | 0.5656 | 0.452 | 1.1 |
| 6 | rs9396681 | 16601068 | C | 0.08816 | 0.1026 | T | 0.9225 | 0.3368 | 0.8453 |
| 6 | rs9350008 | 16601842 | G | 0.3206 | 0.3457 | A | 1.091 | 0.2962 | 0.8932 |
| 6 | rs235158 | 16608839 | A | NA | 0.1483 | C | NA | NA | NA |
| 6 | rs2237185 | 16636650 | A | 0.1476 | 0.1723 | G | 1.724 | 0.1891 | 0.8317 |
| 6 | rs12529351 | 16655600 | A | 0.1578 | 0.1445 | C | 0.5314 | 0.466 | 1.109 |
| 6 | rs236949 | 16676152 | G | 0.2726 | 0.3089 | C | 2.42 | 0.1198 | 0.8385 |
| 6 | rs2237175 | 16681514 | G | 0.1702 | 0.1931 | A | 1.349 | 0.2455 | 0.8572 |
| 6 | rs236970 | 16692361 | A | 0.08158 | 0.1272 | C | 8.556 | 0.003444 | 0.6092 |
| 6 | rs6931635 | 16706112 | C | 0.2739 | 0.2923 | T | 0.6365 | 0.425 | 0.9134 |
| 6 | rs1997716 | 16708658 | T | NA | 0.413 | C | NA | NA | NA |
| 6 | rs2237167 | 16754457 | C | 0.3895 | 0.3953 | A | 0.05551 | 0.8137 | 0.9757 |
| 6 | rs909786 | 16767074 | C | 0.113 | 0.1244 | T | 0.4664 | 0.4946 | 0.8974 |
| 6 | rs719316 | 16780739 | G | 0.3302 | 0.3207 | A | 0.1579 | 0.6911 | 1.045 |
| 6 | rs3812194 | 16788014 | G | 0.324 | 0.3342 | A | 0.18 | 0.6714 | 0.9549 |
| 6 | rs1570487 | 16791838 | T | 0.287 | 0.2802 | C | 0.08808 | 0.7666 | 1.034 |
| 6 | rs1570488 | 16791909 | T | 0.3418 | 0.3466 | C | 0.04052 | 0.8405 | 0.9786 |
| 6 | rs7739138 | 16809155 | G | 0.4375 | 0.4743 | A | 2.087 | 0.1486 | 0.8621 |
| 6 | rs1262918 | 16822596 | G | 0.262 | 0.2705 | A | 0.1431 | 0.7053 | 0.9572 |
| 6 | rs1150628 | 16825195 | A | 0.377 | 0.3719 | G | 0.04054 | 0.8404 | 1.022 |
| 6 | rs3828868 | 16832860 | C | 0.3575 | 0.3805 | T | 0.8678 | 0.3516 | 0.9061 |
| 6 | rs1144695 | 16850744 | C | 0.1478 | 0.1504 | T | 0.02091 | 0.885 | 0.9795 |
| 6 | rs639621 | 16852244 | G | 0.4785 | 0.4949 | T | 0.4076 | 0.5232 | 0.9366 |
| 6 | rs10456788 | 16858835 | G | 0.1166 | 0.117 | C | 0.0004384 | 0.9833 | 0.9967 |
| 6 | rs2301594 | 16862345 | C | 0.4122 | 0.401 | T | 0.199 | 0.6555 | 1.048 |
| 6 | rs13220654 | 17726484 | T | 0.07763 | 0.08616 | C | 0.3691 | 0.5435 | 0.8927 |
| 6 | rs4716165 | 17726502 | C | 0.1376 | 0.1344 | A | 0.03332 | 0.8552 | 1.028 |
| 6 | rs4716167 | 17735569 | A | 0.4363 | 0.4381 | G | 0.005062 | 0.9433 | 0.9927 |
| 6 | rs942467 | 17737595 | G | 0.2765 | 0.2539 | A | 0.9853 | 0.3209 | 1.122 |
| 6 | rs10949436 | 17787686 | G | 0.3 | 0.3103 | C | 0.1861 | 0.6662 | 0.9526 |
| 6 | rs7341276 | 17905586 | A | 0.2164 | 0.1992 | G | 0.6847 | 0.408 | 1.11 |
| 6 | rs733697 | 17916300 | A | 0.4105 | 0.3956 | G | 0.3547 | 0.5515 | 1.064 |
| 6 | rs9396812 | 17953140 | A | 0.4784 | 0.4923 | G | 0.2917 | 0.5891 | 0.9461 |
| 6 | rs4716195 | 17954930 | C | 0.2321 | 0.25 | T | 0.6699 | 0.4131 | 0.9067 |
| 6 | rs2067849 | 18014308 | T | 0.1883 | 0.1941 | C | 0.08068 | 0.7764 | 0.9636 |
| 6 | rs4716200 | 18018304 | G | 0.4286 | 0.4126 | C | 0.4016 | 0.5263 | 1.068 |
| 6 | rs2859778 | 18246806 | A | 0.152 | 0.1684 | C | 0.7579 | 0.384 | 0.8851 |
| 6 | rs3476 | 18333152 | A | 0.2626 | 0.2558 | G | 0.09369 | 0.7595 | 1.036 |
| 6 | rs214511 | 18347766 | T | 0.3351 | 0.3235 | C | 0.2327 | 0.6296 | 1.054 |
| 6 | rs1047033 | 19948471 | G | 0.1957 | 0.199 | A | 0.02579 | 0.8724 | 0.9796 |
| 6 | rs6456349 | 20512998 | G | 0.3607 | 0.367 | A | 0.06509 | 0.7986 | 0.9733 |
| 6 | rs9460501 | 20522665 | G | 0.4299 | 0.4807 | A | 3.993 | 0.04568 | 0.8145 |
| 6 | rs6456353 | 20568249 | A | 0.04704 | 0.0527 | G | 0.2565 | 0.6126 | 0.8874 |
| 6 | rs9295471 | 20576614 | T | 0.4209 | 0.4165 | C | 0.03111 | 0.86 | 1.018 |
| 6 | rs9379382 | 22397440 | T | 0.3713 | 0.3248 | C | 3.642 | 0.05635 | 1.228 |
| 6 | rs849885 | 22399517 | G | 0.1425 | 0.1263 | A | 0.8524 | 0.3559 | 1.149 |
| 6 | rs3804320 | 24285902 | G | 0.377 | 0.3737 | T | 0.01763 | 0.8944 | 1.014 |
| 6 | rs1770909 | 24330431 | T | 0.4173 | 0.4178 | C | 0.0002765 | 0.9867 | 0.9983 |
| 6 | rs4052671 | 24331558 | G | 0.1446 | 0.1298 | A | 0.7035 | 0.4016 | 1.133 |
| 6 | rs793857 | 24353402 | G | 0.3674 | 0.336 | A | 1.605 | 0.2052 | 1.147 |
| 6 | rs9295618 | 24356599 | C | 0.3997 | 0.4109 | T | 0.1963 | 0.6577 | 0.9549 |
| 6 | rs11754278 | 24387055 | A | 0.1561 | 0.1408 | G | 0.7046 | 0.4012 | 1.128 |
| 6 | rs807722 | 24387914 | C | 0.208 | 0.2018 | G | 0.08888 | 0.7656 | 1.039 |
| 6 | rs9356933 | 24429192 | T | 0.1878 | 0.1928 | C | 0.06088 | 0.8051 | 0.9681 |
| 6 | rs1923168 | 24447277 | T | 0.1816 | 0.1974 | C | 0.6234 | 0.4298 | 0.9021 |
| 6 | rs6922023 | 24456096 | A | 0.3971 | 0.3595 | G | 2.301 | 0.1293 | 1.173 |
| 6 | rs6925475 | 24562658 | A | 0.1995 | 0.1918 | G | 0.1409 | 0.7074 | 1.05 |
| 6 | rs2793434 | 24568658 | C | 0.3605 | 0.3651 | T | 0.03399 | 0.8537 | 0.9805 |
| 6 | rs2793444 | 24571476 | A | 0.4339 | 0.435 | G | 0.002035 | 0.964 | 0.9953 |
| 6 | rs9358768 | 24579372 | A | 0.1064 | 0.1021 | G | 0.0732 | 0.7867 | 1.047 |
| 6 | rs2760139 | 24589278 | A | 0.22 | 0.2061 | C | 0.4317 | 0.5112 | 1.086 |
| 6 | rs2744572 | 24598490 | C | 0.4102 | 0.4244 | T | 0.3116 | 0.5767 | 0.9432 |
| 6 | rs7775073 | 24607530 | G | 0.1778 | 0.1592 | A | 0.9331 | 0.3341 | 1.143 |
| 6 | rs2760118 | 24611569 | T | 0.1781 | 0.1601 | C | 0.8775 | 0.3489 | 1.137 |
| 6 | rs3765311 | 24611841 | T | 0.4127 | 0.414 | G | 0.002727 | 0.9583 | 0.9946 |
| 6 | rs2760117 | 24612168 | T | 0.1706 | 0.1521 | C | 0.9579 | 0.3277 | 1.147 |
| 6 | rs2817220 | 24613200 | A | 0.1016 | 0.09656 | G | 0.107 | 0.7436 | 1.058 |
| 6 | rs2143083 | 24615033 | C | 0.455 | 0.4194 | T | 1.939 | 0.1638 | 1.156 |
| 6 | rs4646838 | 24615498 | C | 0.04509 | 0.03968 | T | 0.2722 | 0.6018 | 1.143 |
| 6 | rs2328824 | 24615740 | A | 0.4235 | 0.422 | G | 0.003608 | 0.9521 | 1.006 |
| 6 | rs2760138 | 24620816 | G | 0.4655 | 0.4749 | A | 0.1325 | 0.7159 | 0.9632 |
| 6 | rs2252525 | 24622548 | A | 0.4653 | 0.4577 | C | 0.08892 | 0.7656 | 1.031 |
| 6 | rs17374923 | 24625684 | C | 0.0172 | 0.007937 | T | 2.612 | 0.1061 | 2.187 |
| 6 | rs807515 | 24627865 | G | 0.4761 | 0.4774 | T | 0.00242 | 0.9608 | 0.9949 |
| 6 | rs807517 | 24632683 | T | 0.1003 | 0.09921 | C | 0.004714 | 0.9453 | 1.012 |
| 6 | rs807518 | 24634222 | A | 0.4881 | 0.5 | C | 0.2143 | 0.6434 | 0.9535 |
| 6 | rs809419 | 24634969 | A | 0.3219 | 0.3432 | G | 0.766 | 0.3815 | 0.9086 |
| 6 | rs12192905 | 24639241 | A | 0.007916 | 0.009259 | G | 0.08028 | 0.7769 | 0.8537 |
| 6 | rs12199955 | 24639429 | G | 0.2447 | 0.2779 | T | 2.155 | 0.1421 | 0.8418 |
| 6 | rs12211574 | 24640391 | A | 0.006614 | 0.01058 | G | 0.6983 | 0.4034 | 0.6225 |
| 6 | rs2744597 | 24641053 | A | 0.4718 | 0.4775 | G | 0.04722 | 0.828 | 0.9778 |
| 6 | rs1054899 | 24642172 | A | 0.18 | 0.1614 | C | 0.9227 | 0.3368 | 1.141 |
| 6 | rs2817232 | 24646092 | G | 0.4288 | 0.4444 | A | 0.3785 | 0.5384 | 0.9382 |
| 6 | rs12196913 | 24653286 | C | 0.2196 | 0.2474 | T | 1.63 | 0.2017 | 0.8561 |
| 6 | rs807530 | 24653918 | C | 0.2593 | 0.2368 | G | 1.025 | 0.3114 | 1.128 |
| 6 | rs2817245 | 24655585 | A | 0.1074 | 0.09921 | G | 0.2754 | 0.5997 | 1.093 |
| 6 | rs2744542 | 24666654 | C | 0.1005 | 0.09456 | A | 0.1547 | 0.6941 | 1.07 |
| 6 | rs807525 | 24684016 | G | 0.1039 | 0.1077 | A | 0.05704 | 0.8112 | 0.9612 |
| 6 | rs807509 | 24690011 | C | 0.275 | 0.2823 | G | 0.1012 | 0.7504 | 0.9642 |
| 6 | rs16889511 | 24714015 | G | 0.192 | 0.1748 | C | 0.7542 | 0.3852 | 1.122 |
| 6 | rs761101 | 24740511 | G | 0.1184 | 0.125 | A | 0.1562 | 0.6927 | 0.9403 |
| 6 | rs7766230 | 24741408 | G | 0.3042 | 0.3193 | A | 0.3984 | 0.5279 | 0.9323 |
| 6 | rs2294688 | 24761525 | C | 0.4894 | 0.4698 | G | 0.5793 | 0.4466 | 1.081 |
| 6 | rs12210098 | 26071945 | T | 0.1013 | 0.109 | C | 0.2399 | 0.6243 | 0.9218 |
| 6 | rs2071303 | 26199315 | T | 0.3566 | 0.365 | C | 0.1193 | 0.7298 | 0.964 |
| 6 | rs4634439 | 26705983 | C | 0.08886 | 0.06152 | T | 4.084 | 0.04329 | 1.488 |
| 6 | rs2267633 | 29678820 | G | 0.1771 | 0.2678 | A | 16.65 | 4.49E-05 | 0.5886 |
| 6 | rs2076483 | 29679525 | G | 0.179 | 0.2721 | A | 17.2 | 3.36E-05 | 0.5833 |
| 6 | rs29230 | 29684372 | G | 0.1816 | 0.2657 | A | 14.46 | 0.0001433 | 0.6131 |
| 6 | rs29232 | 29719410 | C | 0.5 | 0.457 | T | 2.572 | 0.1088 | 1.188 |
| 6 | rs3129055 | 29778240 | G | 0.3583 | 0.3357 | A | 0.8062 | 0.3693 | 1.105 |
| 6 | rs9258122 | 29779719 | A | 0.3579 | 0.33 | G | 1.223 | 0.2688 | 1.132 |
| 6 | rs2517713 | 30026078 | G | 0.276 | 0.3632 | T | 12.01 | 0.0005301 | 0.6683 |
| 6 | rs2975042 | 30028516 | G | 0.2824 | 0.3642 | T | 10.43 | 0.001241 | 0.6871 |
| 6 | rs9260734 | 30040645 | A | 0.2535 | 0.3156 | G | 6.688 | 0.009704 | 0.7365 |
| 6 | rs3869062 | 30042870 | G | 0.2333 | 0.2912 | A | 6.06 | 0.01383 | 0.7409 |
| 6 | rs5009448 | 30048467 | T | 0.2977 | 0.3627 | C | 6.572 | 0.01036 | 0.7446 |

Note:

CHR Chromosome

SNP SNP ID

BP Physical position (base-pair)

A1 Minor allele name (based on whole sample)

F_A Frequency of this allele in cases

F_U Frequency of this allele in controls

A2 Major allele name

CHISQ Basic allelic test chi-square (1df)

P Asymptotic p-value for this test

OR Estimated odds ratio (for A1, i.e. A2 is reference)
