# Supplementary material for: Identification of Genes with Allelic Imbalance on 6p Associated with Nasopharyngeal Carcinoma in Southern Chinese
Source: PLoS One. 2011 Jan 20;6(1):e14562. doi: 10.1371/journal.pone.0014562 (PMC3024318; doi:10.1371/journal.pone.0014562)
Supplement: Table S2 — Candidate genes located in “hot spots” identified by meta-analysis (6p21.2-p23). (0.01 MB DOCX) [file pone.0014562.s002.docx]

Supplementary Table 2 Candidate genes located in “hot spots” identified by meta-analysis (6p21.2-p23)

| **Rank** | **Gene Symbol** | **Cytogentic Map** |
| --- | --- | --- |
| 1 | EDN1 | 6p24.1 |
| 2 | ALDH5A1 | 6p22.2-p22.3 |
| 3 | MAK | 6p24 |
| 4 | LY86 | 6p25.1 |
| 5 | CAP2 | 6p22.3 |
| 6 | F13A1 | 6p25.3-p24.3 |
| 7 | DCDC2 | 6p22.1 |
| 8 | HIVEP1 | 6p24-p22.3 |
| 9 | RREB1 | 6p25 |
| 10 | TFAP2A | 6p24 |
| 11 | RIOK1 | 6p24.3 |
| 12 | ATXN1 | 6p23 |
| 13 | TMEM14C | 6p24.2 |
| 14 | SLC35B3 | 6p24.3 |
| 15 | GCNT2 | 6p24.2 |
